# TGF‐β1 improves mucosal IgA dysfunction and dysbiosis following intestinal ischaemia–reperfusion in mice

**DOI:** 10.1111/jcmm.12789

**Published:** 2016-01-28

**Authors:** Xu‐Yu Zhang, Zi‐Meng Liu, Hu‐Fei Zhang, Yun‐Sheng Li, Shi‐Hong Wen, Jian‐Tong Shen, Wen‐Qi Huang, Ke‐Xuan Liu

**Affiliations:** ^1^ Department of AnesthesiologyThe First Affiliated HospitalSun Yat‐sen UniversityGuangzhouChina; ^2^ Surgical Intensive Care UnitThe First Affiliated HospitalSun Yat‐sen UniversityGuangzhouChina

**Keywords:** immunoglobulin A, class switching, mucosal immunity, microbiota, ischemia–reperfusion injury

## Abstract

Intestinal ischaemia/reperfusion (I/R) severely disrupts gut barriers and leads to high mortality in the critical care setting. Transforming growth factor (TGF)‐β1 plays a pivotal role in intestinal cellular and immune regulation. However, the effects of TGF‐β1 on intestinal I/R injury remain unclear. Thus, we aimed to investigate the effects of TGF‐β1 on gut barriers after intestinal I/R and the molecular mechanisms. Intestinal I/R model was produced in mice by clamping the superior mesenteric artery for 1 hr followed by reperfusion. Recombinant TGF‐β1 was intravenously infused at 15 min. before ischaemia. The results showed that within 2 hrs after reperfusion, intestinal I/R disturbed intestinal immunoglobulin A class switch recombination (IgA CSR), the key process of mucosal IgA synthesis, and resulted in IgA dysfunction, as evidenced by decreased production and bacteria‐binding capacity of IgA. Meanwhile, the disruptions of intestinal microflora and mucosal structure were exhibited. Transforming growth factor‐β1 activated IgA CSR as evidenced by the increased activation molecules and IgA precursors. Strikingly, TGF‐β1 improved intestinal mucosal IgA dysfunction, dysbiosis and epithelial damage at the early stage after reperfusion. In addition, SB‐431542, a specific inhibitor of activating mothers against decapentaplegic homologue (SMAD) 2/3, totally blocked the inductive effect of TGF‐β1 on IgA CSR and almost abrogated the above protective effects on intestinal barriers. Taken together, our study demonstrates that TGF‐β1 protects intestinal mucosal IgA immunity, microbiota and epithelial integrity against I/R injury mainly through TGF‐β receptor 1/SMAD 2/3 pathway. Induction of IgA CSR may be involved in the protection conferred by TGF‐β1.

## Introduction

Intestinal ischaemia/reperfusion (I/R) is a grave condition during haemorrhagic or septic shock, severe trauma and burn, abdominal aortic surgery and cardiopulmonary bypass, and it usually results in high morbidity and mortality in the critical setting 1. A latest multicenter study showed that acute mesenteric ischaemia in 780 patients was associated with a 58% death rate 2. We previously demonstrated that intestinal mucosal structure was severely damaged following intestinal I/R 3, 4. In addition, several studies showed that intestinal I/R also disrupted mucosal immunity and microflora 5, 6. The impaired intestinal mechanical, immune and biological barriers lead to bacterial translocation, and subsequently gut‐origin sepsis and multiple organ dysfunction syndrome 7, 8. To date, however, few studies have investigated the mechanism and therapy for the immune disorder and dysbiosis caused by intestinal I/R injury.

Gut mucosal immunoglobulin (Ig) A is critical for not only defending the host against pathogens but also regulating the host‐commensal relationship 9, 10. However, we recently demonstrated that intestinal I/R resulted in impaired class switch recombination (CSR) of IgM B^+^ cells, a key biological process involved in mucosal IgA synthesis, in peyer's patches (PPs) and decreased secretory IgA (sIgA) concentration in the gut lumen at 2 hrs after reperfusion 11. Transforming growth factor (TGF)‐β1, a polypeptide member of the TGF‐β superfamily, plays a pivotal role in cellular proliferation, differentiation and apoptosis as well as mucosal inflammation and immunity 12, 13. Importantly, TGF‐β1 can potently and independently activate IgA CSR and promote IgA‐secreting plasma cells 14. Therefore, we hypothesized that TGF‐β1 administration may increase IgA synthesis by inducing IgA CSR and subsequently improve gut mucosal dysbiosis and histological injury following intestinal I/R.

Taken together, this study was designed to investigate the effects of TGF‐β1 on intestinal mucosal immunity, microflora and histology after intestinal I/R and to explore the potential signalling and mechanisms.

## Materials and methods

### Animals and operative procedure

The current experiment was approved by the Animal Care Committee of Sun Yat‐sen University and was performed in accordance with National Institutes of Health guidelines for the experimental animals. Eight‐ to ten‐week‐old SPF male Balb/c mice (28.6–32.3 g) were housed in individual cages and acclimated for 1 week before protocol entry.

Mice were anaesthetized with intraperitoneally pentobarbital (70 mg/kg at induction and 30 mg/kg at 1 hr later) and ketamine (50 mg/kg at induction). The small intestine was exteriorized by midline laparotomy, and the intestinal I/R model was established by occluding the superior mesenteric artery (SMA) with a microvessel clip for 1 hr, as we described previously 3, 4. After 1 hr of occlusion, the clip was removed and abdominal incision was sutured. During the study period, all procedures were performed on spontaneously breathing animals, and body temperature was maintained at about 36°C with the aid of a heating pad.

### Groups and drug administration

**Figure 1:**
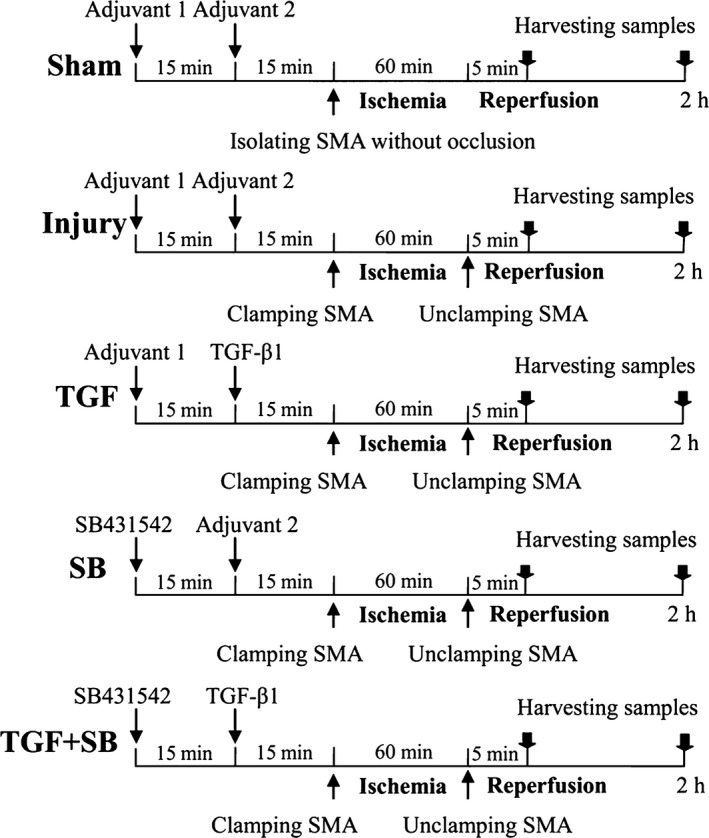
The experimental protocol of groups and drugs. Sham group: adjuvants were given and SMA was isolated without occlusion; injury group: adjuvants were given and SMA was occluded for 60 min.; TGF group: 1 μg TGF‐β1 was dissolved in adjuvant 2 and then injected at 15 min. before ischaemia; SB group: 0.5 mg SB‐431542 was dissolved in adjuvant 1 and then administrated at 30 min. before ischaemia; TGF+SB group: the same dose of TGF‐β1 and SB‐431542 was administrated at corresponding time‐point, respectively. Adjuvant 1 = 10% DMSO 100 μl; adjuvant 2 = 100 μl mixture (10 mM citric acid 10 μl and PBS containing 5% trehalose 90 μl); SMA: superior mesenteric artery; DMSO: dimethyl sulfoxide; TGF: transforming growth factor; SB: SB‐431542, specific inhibitor of SMAD 2/3 transcription.

The current experimental protocol was determined according to previous literatures 15, 16 and our preliminary results. The mice were randomly allocated into five groups. Sham group (sham): the animals received adjuvants and underwent laparotomy without occlusion of the SMA. I/R group (injury): the animals received adjuvants, and the SMA was clamped for 1 hr. Transforming growth factor‐β1 group (TGF): 1 μg (100 μl) recombinant human TGF‐β1 (PeproTech Inc, Rocky Hill, NJ, USA) was infused through caudal vein at 15 min. before ischaemia. Transforming growth factor‐β1 inhibitor group (SB): 0.5 mg SB‐431542 was dissolved in 100 μl 10% dimethyl sulfoxide and injected intraperitoneally at 30 min. before ischaemia. Transforming growth factor‐β1 + SB‐431542 group (TGF+SB): isometric TGF‐β1 and SB‐431542 was administrated at corresponding time‐point, respectively. The detailed experimental protocol was shown in Figure 1.

### Biological samples collection

After euthanizing the mice, a ~0.5‐cm segment of intestine was cut from 10 cm to terminal ileum for immunohistochemical analysis. PP samples were carefully excised for isolating lymphocytes 5. Then, the intestine was opened longitudinally and the faecal contents were collected for detecting the bacteria‐binding capacity of IgA. A ~10‐cm intestinal fragment next to ileocecal valve was flushed with 3‐ml chilled PBS, and the washings were collected for detecting sIgA concentration. Then, this fragment was cut into small pieces for isolating lymphocytes from lamina propria (LP) 5. Another segment of small intestine (~10 cm) was obtained from 12 cm to terminal ileum, and the intestinal mucosa was scraped off gently and preserved at liquid nitrogen for detecting IgA mRNA expression 17. Finally, caecal faeces were collected for analysing gut microbiota. For each parameter, 5–6 samples per group were collected and analysed.

### Immunohistochemical analysis

The small intestine segment was fixed in paraformaldehyde and embedded in paraffin. The fixed tissues were sectioned and stained with haematoxylin–eosin. Two independent pathologists assessed the histological damage by using Chiu's score 4.

Moreover, the sections were subjected to deparaffinization and rehydration and then stained with FITC‐conjugated antimouse IgA diluted 1/100 for 1 hr. Finally, nuclear staining was accomplished with 4′, 6‐diamidino‐2‐phenylindole. The stained slides were examined with a fluorescence microscope (Nikon, Tokyo, Japan).

### Cells isolation from PP and LP

Cells suspension of PP and LP was prepared according to the modified method descried previously 5. Briefly, PPs were pressed through a steel mesh grid. The PP fragments were incubated with collagenase VIII (40 U/ml) in RPMI 1640. The cell suspensions were passed through 100‐μm nylon filters.

The small pieces of intestine were incubated in PBS containing ethylenediaminetetraacetic acid‐Na_2_, dithiothreitol and foetal bovine serum (FBS). Supernatants containing epithelial and intraepithelial cells were removed. The remaining tissues were incubated with RPMI 1640 containing collagenase VIII (100 U/ml), 5% FBS and a 1% antibiotic mixture. Supernatants were filtered through nylon filters and centrifuged. The cell pellets were resuspended in 40% Percoll and then overlaid with 80% Percoll. After centrifugation, lymphocytes from LP were aspirated from the interface of 40/80% Percoll and diluted with RPMI 1640. Viable cells were counted by using trypan blue dye exclusion.

### Quantitative real‐time PCR and flow cytometry

By using quantitative real‐time PCR (qRT‐PCR) and flow cytometric analysis, mRNA expression of biomarkers and percentage of IgA^+^/IgM^+^B220^+^ cells were applied to investigate IgA class switching.

Total RNA from the cells and tissues was isolated by using TRIzol reagent according to the manufacturer's protocol. The qRT‐PCR was performed on a C1000 Touch Thermal cycler with SYBR Green. Transforming growth factor‐β1 and activation‐induced cytidine deaminase are essential to IgA CSR, and germline α transcripts and Iμ‐Cα circle transcripts are the representative activation biomarkers for IgA switching 17. The primers for these four biomarkers and IgA were determined based on the previous data 18, 19, 20, 21 (Table 1). The relative amounts of mRNA transcripts were calculated by using the standard curve method and normalized by control GAPDH.

**Table 1:** Primer sets used for quantitative real‐time PCR

Gene	Forward primer 5′–3′	Reverse primer 5′–3′
TGF‐β1	ctgtagcccacgtcgtagc	ttgagatccatgccgttg
AID	cgtggtgaagaggagagatagtg	cagtctgagatgtagcgtaggaa
GLTα	caagaaggagaaggtgattcag	gagctggtgggagtgtcagtg
αCTs	ccaggcatggttgagatagagatag	aatggtgctgggcaggaagt
IgA (α‐chain)	cgtccaagaattggatgtga	agtgacaggctgggatgg
GADPH	tgtgtccgtcgtggatctga	cctgcttcaccaccttcttgat

Flow cytometric analysis was performed as previously described 5. Briefly, the cell pellets obtained from PP and LP were resuspended. The lymphocytes were stained with 1 μg/ml antimouse IgA, antimouse IgM and antimouse B220 to identify IgA^+^ and IgM^+^B220^+^ cells, respectively. The cells were detected with a FACSCalibur flow cytometer. The data were analysed by Flowjo software.

### Detection of sIgA in intestinal lavage

Secretory IgA concentration was detected by using the ELISA kit (Cusabio, Wuhan, China), as described previously 18. Briefly, the intestinal washing was centrifuged at 1250 g for 20 min., and the supernatant was harvested to measure the sIgA concentration.

### Gut bacterial community analysis

Gut microbiota was analysed by a 16S rRNA gene sequencing on the Illumina MiSeq platform according to the protocol of Miseq system. Briefly, bacteria genomic DNA was extracted from 0.5 g caecal faeces by using PowerSoil^®^ DNA Isolation Kit (MO BIO Laboratories, Carlsbad, CA, USA). The primers of V4 hypervariable regions were used to amplify templates from genomic DNA. Data analysis was performed by Mothur software (Version 1.28, http://www.mothur.org/, USA).

### Evaluation of intestinal bacteria coated with IgA

Flow cytometric analysis of IgA‐binding bacteria was performed as previously described 19. Briefly, the faeces were suspended in PBS and centrifuged. Then, supernatant was centrifuged at 5650 g to remove non‐bound Igs. The pellets were resuspended in 1 ml of FBS/PBS (1% w/v). Bacteria were stained with antimouse IgA. Finally, bacteria pellets were resuspended in 4 μg/ml propidium iodide (PI)/PBS and analysed by FACScalibur. All events that stained with PI were regarded as bacteria. The percentage of bacteria coated with IgA was calculated as: the number of PI^+^ IgA^+^ cells/the number of total PI^+^ cells×100.

### Survival analysis

The survival analysis was performed in independent mice as we previously described 4. Briefly, the mice were managed with the same interventions and then transferred to their cages. Each mouse was monitored *via* video recording for 24 hrs.

### Statistical analysis

The data were analysed with SPSS 15.0 software (SPSS Inc, Chicago, IL, USA). Survival time after reperfusion was expressed as median (95% confidence intervals), and results were compared by Kaplan–Meier log‐rank test. The other data were expressed as mean ± S.E., and one‐way anova (Tukey post‐test) was used for comparisons. *P* < 0.05 in two‐tailed testing was considered statistically significant.

## Results

### The effects of TGF‐β1 on IgA CSR following intestinal I/R

**Figure 2:**
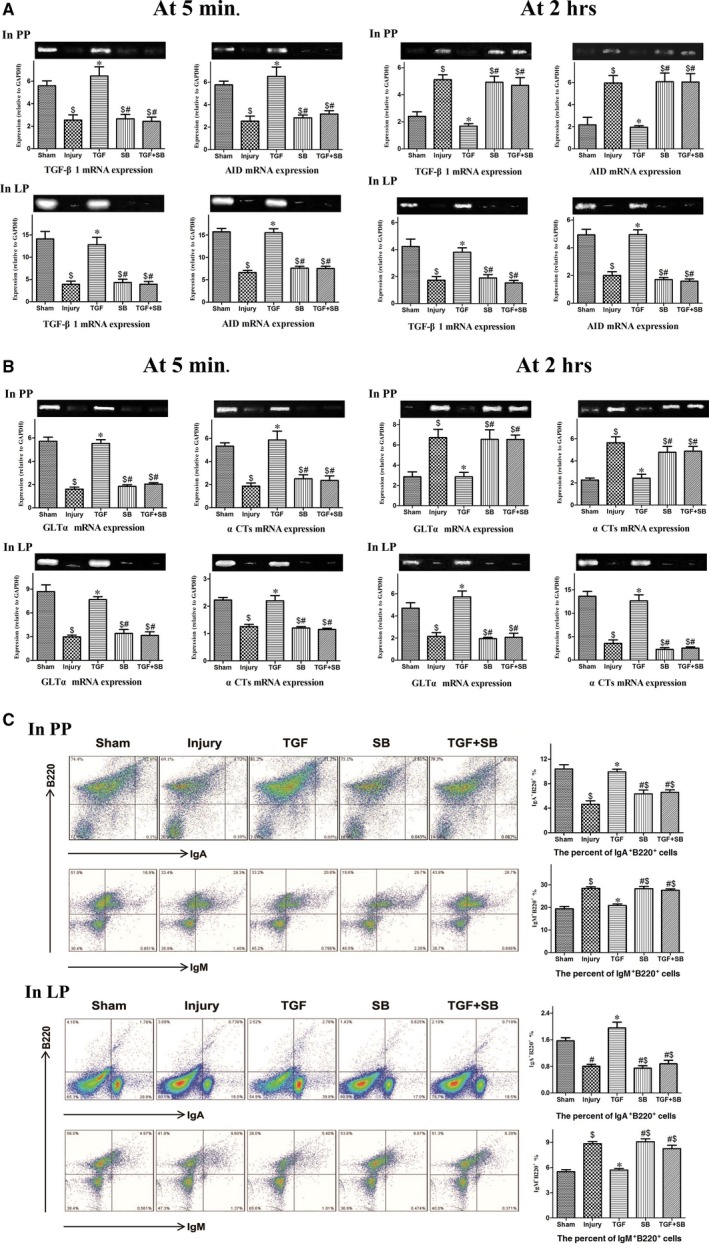
Changes of IgA CSR in PP and LP following intestinal I/R. (**A**) Changes of TGF‐β1 and AID in PP and LP at 5 min. and 2 hrs after reperfusion. Analysis of relative mRNA expression of these two biomarkers was shown, respectively. And representative blots were presented on the top. (**B**) Changes of GLTα and αCTs in PP and LP at 5 min. and 2 hrs after reperfusion. (**C**) Changes of IgA^+^B220^+^ and IgM^+^B220^+^ cells from PP and LP at 2 hrs after reperfusion. Representative flow cytometric profiles were presented in the left panel. Analysis of the percentage of IgA^+^ and IgM^+^B220^+^ cells was shown in the right. Data are expressed as mean ± S.E., *n* = 5 or 6. Results were compared by anova with Tukey post‐test. ^$^ *P* < 0.01 *versus* the sham group; *P < 0.01 *versus* the injury group; ^#^ *P* < 0.01 *versus* the TGF group. Ig: immunoglobulin; TGF‐β1: transforming growth factor‐β1; AID: activation‐induced cytidine deaminase; CSR: class switch recombination; GLTα: germline α transcripts; αCTs: Iμ‐Cα circle transcripts; PP: peyer's patches; LP: lamina propria.

At 5 min. after reperfusion, the mRNA expression of all molecular biomarkers in PP and LP was lower in the injury group than that in the sham group (all *P* < 0.01). At 2 hrs after reperfusion, in the injury group, the expression of all molecules in PP was elevated but the expression in LP was lower (all *P* < 0.01 *versus* sham; Fig. 2A and B). Transforming growth factor‐β1 increased the expression of all molecules in LP. Whereas, in PP, TGF‐β1 increased the molecular expression at 5 min. after reperfusion (all *P* < 0.01), but decreased the expression at 2 hrs (all *P* < 0.01; Fig. 2A and B).

In the injury group, the percentage of IgA^+^B220^+^ cells decreased and IgM^+^B220^+^ cells increased in PP and LP at 2 hrs after reperfusion (all *P* < 0.01 *versus* sham; Fig. 2C). Transforming growth factor‐β1 restored the balance of IgA^+^ and IgM^+^ B cells in both PP and LP (Fig. 2C).

### The effects of TGF‐β1 on gut mucosal IgA production and bacteria‐binding capacity following intestinal I/R

**Figure 3:**
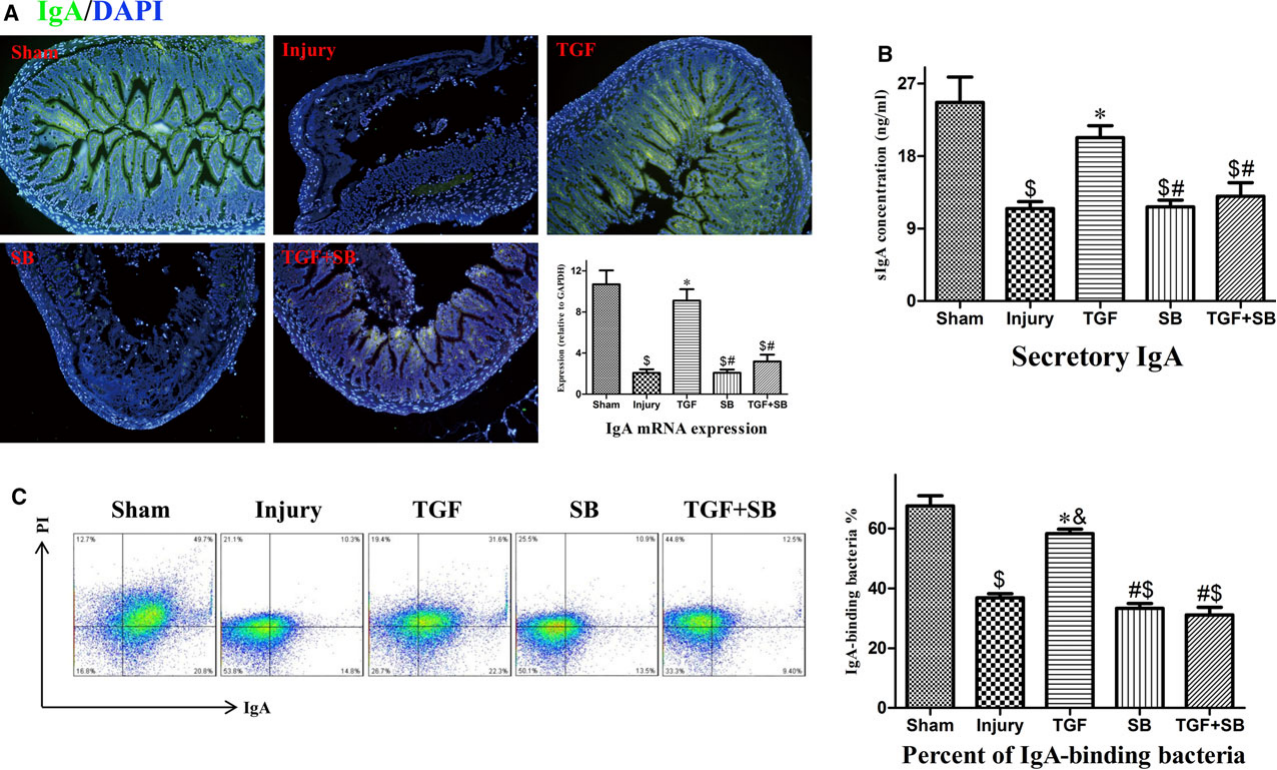
Changes of mucosal IgA production and bacteria‐binding capacity following intestinal I/R. (**A**) Changes of intestinal IgA expression at 2 hrs after reperfusion. Immunofluorescence of mucosal tissues in each group was shown, respectively. The intestinal sections were stained with anti‐IgA (green) and DAPI (blue) counterstained nuclei. Analysis of relative mRNA expression of IgA in the intestinal mucosa was also presented. (**B**) Changes of IgA secretion at 2 hrs after reperfusion. Analysis of secretory IgA concentration in the lavage was shown. (**C**) Changes of IgA‐binding bacteria in the gut lumen at 2 hrs after reperfusion. Representative flow cytometric profiles of IgA^+^ PI ^+^ cells were presented in the left panel. Analysis of the percentage of IgA‐binding bacteria in the faeces was presented in the right. Data are expressed as mean ± S.E., *n* = 5 or 6. Results were compared by anova with Tukey post‐test. ^&^ *P* < 0.05 *versus* the sham group; ^$^ *P* < 0.01 *versus* the sham group; *P < 0.01 *versus* the injury group; ^#^ *P* < 0.01 *versus* the TGF group. IgA: immunoglobulin A; DAPI: 4′,6‐diamidino‐2‐phenylindole; PI: propidium iodide.

IgA mRNA expression in the intestinal mucosa and sIgA concentration in the lavage were lower in the injury group than that in the sham group at 2 hrs after reperfusion (both *P* < 0.001; Fig. 3A and B). Meanwhile, the percentage of bacteria coated with IgA in the faeces was lower in the injury group than that in the sham group (Fig. 3C). Transforming growth factor‐β1 increased IgA production and bacteria‐coating percentage (all *P* < 0.01 *versus* injury; Fig. 3).

### The effects of TGF‐β1 on gut microbiota, histological injury and mortality following intestinal I/R

**Figure 4:**
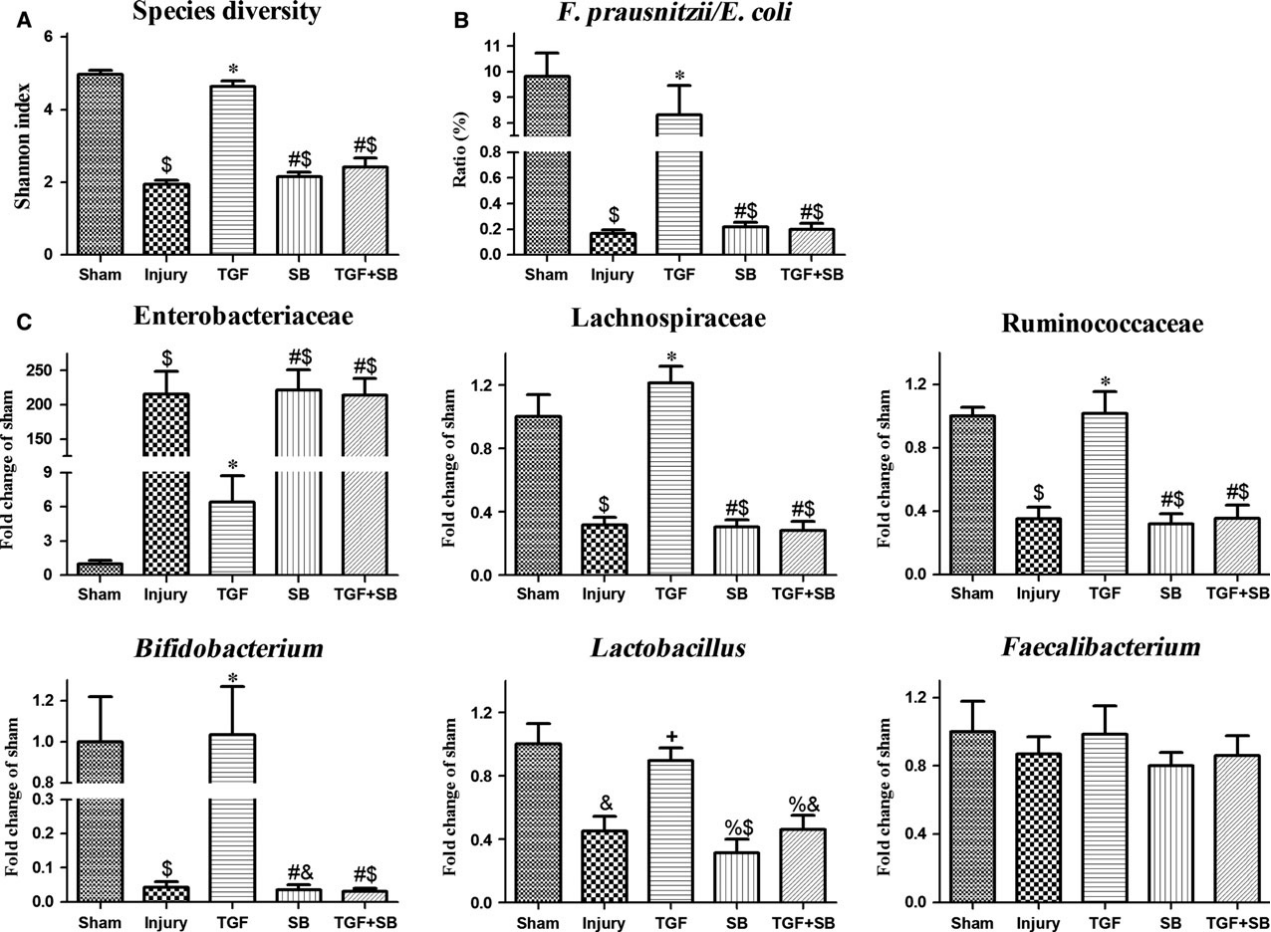
Changes of gut microbiota following intestinal I/R. Changes of colonic bacterial diversity and compositions at 2 hrs after reperfusion were shown, respectively. (**A**) Species diversity. The Shannon index in each group was analysed. (**B**) *Faecalibacterium prausnitzii*/*Escherichia coli* ratio. This usual indicator for gut dysbiosis in each group was analysed. (**C**) Bacterial compositions. The number of bacteria in specific bacterial taxa in each group was analysed. Changes of bacterial quantity were represented by fold change relative to sham mice. Data were expressed as mean ± S.E., *n* = 3 or 4. Results were compared by anova with Tukey post‐test. ^$^ *P* < 0.01 *versus* the sham group; *P < 0.01 *versus* the injury group; ^#^ *P* < 0.01 *versus* the TGF group; ^&^ *P* < 0.05 *versus* the sham group; ^+^ *P* < 0.05 *versus* the injury group; ^%^ *P* < 0.05 *versus* the TGF group.

The species diversity of colonic bacteria was lower in the injury group than that in the sham group (*P* < 0.001; Fig. 4A). In the injury group, *Enterobacteriaceae* increased about 230‐fold, but the obligate anaerobes, *Ruminococcaceae* and *Lachnospiraceae*, and the healthy bacteria, *Lactobacillus* and *Bifidobacterium*, significantly decreased (all *P* < 0.01 *versus* sham; Fig. 4C). *Faecalibacterium* did not significantly change, but the *Faecalibacterium prausnitzii/Escherichia coli* ratio, an indicator of intestinal dysbiosis 22, was lower in the injury group (Fig. 4B). Transforming growth factor‐β1 significantly alleviated the alterations of microbiota (all *P* < 0.05; Fig. 4).

**Figure 5:**
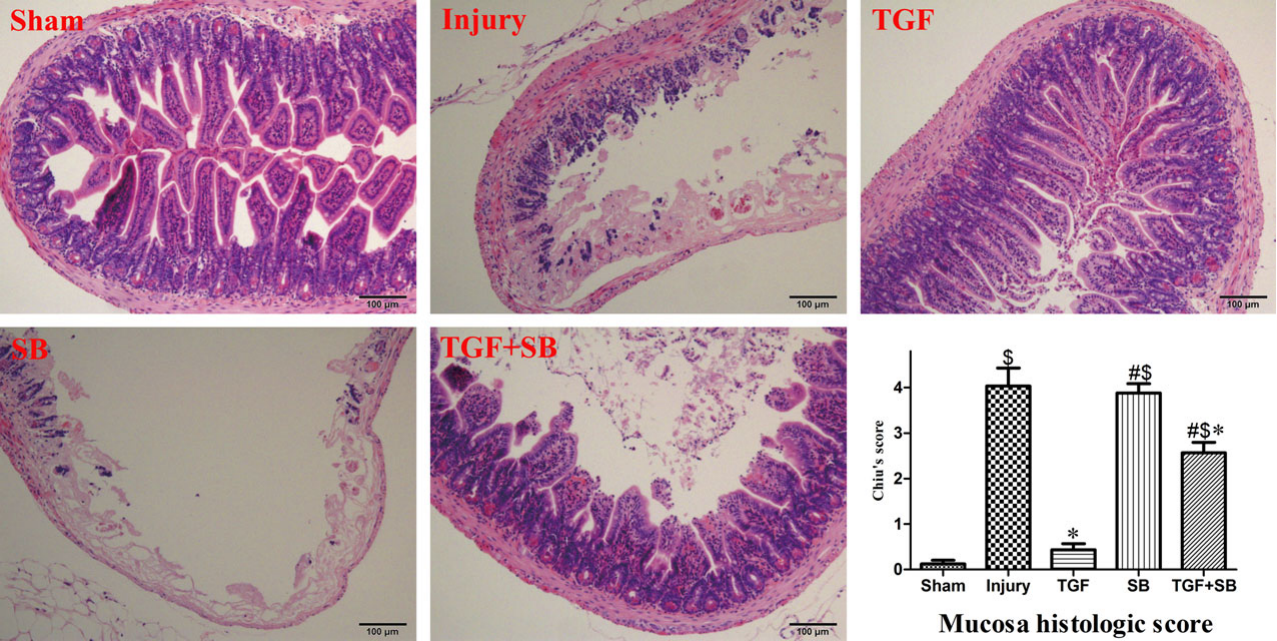
Changes of intestinal mucosal histology. The intestinal sections (×100) were stained with haematoxylin–eosin. In the sham group, the intestinal villi and glands were normal. Severe intestinal histological damage was observed in the injury and SB groups: normal structure of mucosa disappeared, and severe haemorrhage was present. In the TGF group, the mucosal structures were almost normal, and only very slight mucosal sloughing was seen at villi tips. In the TGF+SB group, disintegrated intestinal villi and increased subepithelial gap were observed. Whereas haemorrhage was rarely detected and the mucosal injury was much better than that seen in the injury group. Analysis of Chiu's score in each group was also presented. Data are expressed as mean ± S.E., *n* = 5 or 6. Results were compared by anova with Tukey post‐test. ^$^ *P* < 0.01 *versus* the sham group; *P < 0.01 *versus* the injury group; ^#^ *P* < 0.01 *versus* the TGF group.

Chiu's score was higher in the injury group (*P* < 0.001 *versus* sham). Significant decrease of Chiu's score was detected in the TGF group when compared with the injury group (0.43 ± 0.13 *versus* 4.03 ± 0.39, *P* < 0.001; Fig. 5).

**Figure 6:**
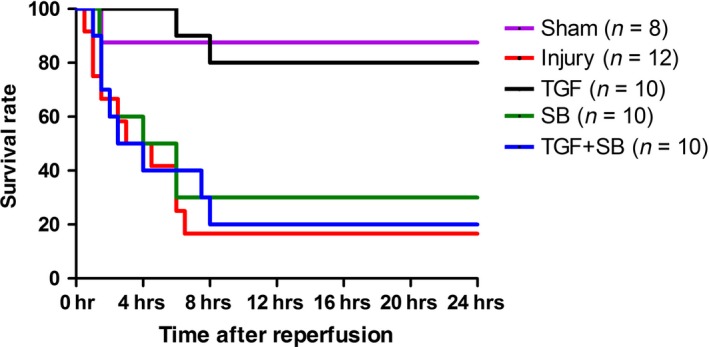
Survival analysis. The independent animals underwent various interventions based on the experimental protocol. Survival time is calculated from the beginning of reperfusion, *n* = 8–12. Results were compared by Kaplan–Meier log‐rank test.

The survival time and mortality rate of the mice in the injury group was 3 hrs (0–6.4 hrs) and 83.3%, respectively (Fig. 6). Transforming growth factor‐β1 dramatically reduced mortality rate to 20% (*P* = 0.004 *versus* injury).

### The effects of SB‐431542 on TGF‐β1‐induced intestinal I/R protection

SB‐431542 alone (SB group) produced no active effects on the above‐mentioned variables in comparison with the injury group (all *P* > 0.05; Figs. 2, 3, 4, 5, 6). On the other hand, SB‐431542 eliminated the effects of recombinant TGF‐β1 on IgA switching biomarkers and IgA^+^ precursor cells (Fig. 2). Moreover, the protective effects of TGF‐β1 on the mucosal IgA, microbiota and survival were totally diminished (Figs 3, 4 and 6). Whereas the Chiu's score in the TGF+SB group (2.57 ± 0.23) was still statistically lower than that in the injury group (*P* = 0.002; Fig. 5).

## Discussion

This study shows that TGF‐β1 provides striking protection for intestinal barriers following intestinal I/R. During perioperative period, manipulation of TGF‐β1 expression through several medical interventions can help the clinicians to prevent and treat intestinal I/R‐related diseases.

Mucosal IgA is essential to maintain homeostasis in the gut lumen. By specifically and non‐specifically binding bacteria, intestinal IgA regulates gut microbiota and maintains mucosal integrity 10, 23. Whereas the present data showed that intestinal I/R reduced not only gut IgA production but also its bacteria‐binding capacity at 2 hrs after reperfusion (Fig. 3). To date, the mechanisms by which intestinal I/R disrupts gut IgA immunity remain unclear. In the organized mucosa‐associated lymphoid tissues, the preferential presence of IgM^+^B220^+^ and IgA^+^B220^+^ cells belongs to pre‐ and post‐IgA isotype class‐switched B cells 20, 24. Therefore, in this study, IgM^+^ and IgA^+^B220^+^ cells as well as four biomarkers were used to explore the change of IgA class switching during intestinal I/R. In the inductive sites (PP) and effector sites (LP) of gut immunity, decreased expression of biomarkers at 5 min. and imbalance of IgA^+^/IgM^+^ B cells at 2 hrs after reperfusion indicate that IgA CSR is inhibited at early reperfusion phase (Fig. 2). Subsequently, impaired IgA CSR results in IgA dysfunction (Fig. 3). In addition, the destruction of gut mucosal tissues (Fig. 5) also disturbs IgA synthesis, transport and secretion. Therefore, the disrupted gut IgA immunity caused by I/R injury deteriorates intestinal dysbiosis and epithelial damage.

The gut microflora was regarded as ‘a forgotten organ’ 25. Actually, pathogens and virulence factors from gut flora are thought to be integral to the development of multiple organ dysfunction syndrome 26, 27. Moreover, enteric dysbiosis increases septic complications and mortality in surgical and critically ill patients 28, 29. Recently, Hayakawa *et al*. reported that the gut flora in critically ill patients altered immediately after a severe insult 30. Through denaturing gradient gel electrophoresis, Wang *et al*. demonstrated that, after 30‐min. occlusion of SMA in rats, colonic *E. coli* dramatically increased within 1 hr and microflora differed significantly at 6 hrs after reperfusion 6. Whereas, in this study, the sequencing data showed that, at 2 hrs after reperfusion, both the obligate anaerobes and healthy bacteria markedly reduced in colonic faeces. Meanwhile, decreased species diversity and *F. prausnitzii/E. coli* ratio were detected (Fig. 4). We suggest that long‐duration ischaemia, dissimilar animals and analytical techniques may be involved in the discrepancies. Breakdown of gut microflora after critical insults increases the risk of pathogens colonization, bacterial translocation, and uncontrolled inflammation and infection 31. Overall, severe disruption of intestinal homeostasis, as evidenced by IgA dysfunction, dysbiosis and injured epithelia, at the early reperfusion phase may lead to the high mortality (Fig. 6). Indeed, survival analysis in previous literatures also showed that most animals underwent intestinal I/R died within several hours after reperfusion 4, 32.

It has been demonstrated that isoflurane, a volatile anaesthetic, decreased epithelial injury after intestinal I/R by upregulating TGF‐β1 expression 33. However, the mechanisms by which TGF‐β1 alleviates intestinal damages under intestinal I/R have not been investigated. Several authors previously demonstrated that induction of IgA CSR by oral clarithromycin could protect against influenza A virus infection in animals and human beings through increasing the production and virus‐neutralizing activities of airway sIgA 34, 35, suggesting that activation of IgA CSR is an effective way to reduce mucosal injury. In general terms, development of IgA generation is a long‐term process 36. However, Yoshiya *et al*. reported that intestinal IgA expression significantly increased at 4 hrs after reperfusion 37. Furthermore, our results also showed that all activation biomarkers for IgA CSR elevated in the inductive sites at 2 hrs after reperfusion (Fig. 2A and B), indicating that, under conditions of intestinal I/R, IgA response initiates within several hours following reperfusion. Based on the results of biomarkers at 5 min. after reperfusion (Fig. 2A and B), we suggest that recombinant TGF‐β1 accelerates the activation of IgA CSR. Thus, at 2 hrs after I/R, although the biomarkers decreased in PP, the use of TGF‐β1 restores the balance of IgA^+^/IgM^+^ B cells in both inductive and effector sites (Fig. 2C) and subsequently diminished IgA dysfunction (Fig. 3). In different gastrointestinal diseases, robust IgA coating can reduce mucosal invasion through accurately identifying and eliminating pathogenic bacteria 38, 39. Also, Suzuki *et al*. reported that reconstitution of normal IgA level by anastomosis restored the normal composition of gut microbiota in IgA‐deficient mouse 40. Therefore, we suggest that the restoration of mucosal IgA induced by TGF‐β1 *via* inducing IgA CSR may attenuate gut dysbiosis and epithelial injury and ultimately reduce mice’ mortality. To the best of our knowledge, this study, for the first time, explores the therapeutic approach for intestinal I/R‐induced mucosal damages by enhancing cellular switching and immune molecular activities. Our findings suggest that, in the clinical setting, inhalational anaesthesia may be appropriate for the patients with potential intestinal I/R. In contrast, morphine should be prohibited in these critically ill patients due to its inhibitory effect on gut TGF‐β expression 41.

Transforming growth factor‐β1 regulates the cellular and immune functions through differential signalling pathways 42, 43. In this study, SB‐431542, a specific inhibitor of TGF beta receptor 1/activating mothers against decapentaplegic homologue (SMAD) 2 and 3 44, was added to explore the protective signalling of TGF‐β1. Because TGF‐β1 induces IgA CSR *via* SMAD 2/3 45, SB‐431542 totally eliminated the activation of IgA CSR and the improvement of IgA responses induced by TGF‐β1 (Figs 2 and 3). Interestingly, SB‐431542 just partly diminished the protective effect of TGF‐β1 on intestinal epithelia (Fig. 5), indicating that TGF‐β1 protected epithelial tissues through SMAD‐dependent and SMAD‐independent pathways and TGF‐β1 improved IgA dysfunction mainly by inducing IgA CSR, but not by preserving mucosal tissues. Therefore, we suggest that, during early reperfusion period, activation of IgA CSR conferred by TGF‐β1 is involved in the improvement of gut mucosal immunity and other damages.

There were several limitations in our experimental design. First, because there is no feasible technique, either *in vivo* or *in vitro*, to exclusively block the process of IgA CSR, the role of IgA CSR in TGF‐β1‐induced intestinal I/R protection was not definitely clarified in this study. Nevertheless, induction of IgA CSR has been proven to be beneficial for mucosal invasion 34, 35. Second, TGF‐β1 has numerous physiological effects. In this study, we focused on investigating its effect of IgA switching in intestinal I/R protection. The exact impacts and mechanisms of TGF‐β1 for intestinal I/R injury need to be further clarified. Third, TGF‐β1 was administrated before ischaemia in this model. However, in the clinical setting, the vast majority of intestinal I/R is diagnosed after or concurrent to the insult. The effects of TGF‐β1 post‐treatment on intestinal barriers need to be explored. Finally, severe intestinal damages commonly take place during the early reperfusion stage. Thus, we just detected the variables within 2 hrs after reperfusion. Prolonged observation is needed to evaluate the long‐term effect of TGF‐β1 on intestinal I/R injury.

In summary, intestinal I/R leads to decreased production and bacteria‐binding capacity of gut mucosal IgA, as well as mucosal dysbiosis and histological injury during the early reperfusion phase. Recombinant TGF‐β1 pretreatment comprehensively improves the above damages mainly through TGF‐β Receptor 1/SMAD 2/3 signalling pathway. The changes of IgA CSR may be involved in the pathogenesis and treatment for intestinal I/R injury. The present study provides new insights into the relation of the gut IgA immunity and mucosal barriers under intestinal I/R insult and exhibits a novel avenue for treating the relevant diseases.

## Conflicts of interest

The authors confirm that there are no conflicts of interest.

## Supporting information


**Data S1** Materials and methods.

## References

[1] Mallick IH , Yang W , Winslet MC , *et al* Ischemia‐reperfusion injury of the intestine and protective strategies against injury. Dig Dis Sci. 2004; 49: 1359–77. 15481305 10.1023/b:ddas.0000042232.98927.91

[2] Leone M , Bechis C , Baumstarck K , *et al* Outcome of acute mesenteric ischemia in the intensive care unit: a retrospective, multicenter study of 780 cases. Intensive Care Med. 2015; 41: 667–76. 25731634 10.1007/s00134-015-3690-8

[3] Liu KX , Chen SQ , Huang WQ , *et al* Propofol pretreatment reduces ceramide production and attenuates intestinal mucosal apoptosis induced by intestinal ischemia/reperfusion in rats. Anesth Analg. 2008; 107: 1884–91. 19020134 10.1213/ane.0b013e3181884bbf

[4] Zhang XY , Liu ZM , Wen SH , *et al* Dexmedetomidine administration before, but not after, ischemia attenuates intestinal injury induced by intestinal ischemia‐reperfusion in rats. Anesthesiology. 2012; 116: 1035–46. 22417965 10.1097/ALN.0b013e3182503964

[5] Fukatsu K , Sakamoto S , Hara E , *et al* Gut ischemia‐reperfusion affects gut mucosal immunity: a possible mechanism for infectious complications after severe surgical insults. Crit Care Med. 2006; 34: 182–7. 16374173 10.1097/01.ccm.0000196207.86570.16

[6] Wang F , Li Q , Wang C , *et al* Dynamic alteration of the colonic microbiota in intestinal ischemia‐reperfusion injury. PLoS ONE. 2012; 7: e42027. 22848694 10.1371/journal.pone.0042027 PMC3407053

[7] MacFie J , O'Boyle C , Mitchell CJ , *et al* Gut origin of sepsis: a prospective study investigating associations between bacterial translocation, gastric microflora, and septic morbidity. Gut. 1999; 45: 223–8. 10403734 10.1136/gut.45.2.223 PMC1727620

[8] Deitch EA . Gut‐origin sepsis: evolution of a concept. Surgeon. 2012; 10: 350–6. 22534256 10.1016/j.surge.2012.03.003 PMC3413774

[9] Pabst O . New concepts in the generation and functions of IgA. Nat Rev Immunol. 2012; 12: 821–32. 23103985 10.1038/nri3322

[10] Strugnell RA , Wijburg OL . The role of secretory antibodies in infection immunity. Nat Rev Microbiol. 2010; 8: 656–67. 20694027 10.1038/nrmicro2384

[11] Zhang XY , Liu ZM , Zhang HF , *et al* Decreased PD‐1/PD‐L1 expression is associated with the reduction in mucosal immunoglobulin A in mice with intestinal ischemia reperfusion. Dig Dis Sci. 2015; 60: 2662–9. 25944714 10.1007/s10620-015-3684-y

[12] Ohtsuka Y , Sanderson IR . Transforming growth factor‐beta: an important cytokine in the mucosal immune response. Curr Opin Gastroenterol. 2000; 16: 541–5. 17031135 10.1097/00001574-200011000-00014

[13] Kajdaniuk D , Marek B , Borgiel‐Marek H , *et al* Transforming growth factor β1 (TGFβ1) in physiology and pathology. Endokrynol Pol. 2013; 64: 384–96. 24186596 10.5603/EP.2013.0022

[14] Endsley MA , Njongmeta LM , Shell E , *et al* Human IgA‐inducing protein from dendritic cells induces IgA production by naive IgD+ B cells. J Immunol. 2009; 182: 1854–9. 19201837 10.4049/jimmunol.0801973 PMC2878093

[15] Ma M , Ma Y , Yi X , *et al* Intranasal delivery of transforming growth factor‐beta1 in mice after stroke reduces infarct volume and increasesneurogenesis in the subventricular zone. BMC Neurosci. 2008; 9: 117. 19077183 10.1186/1471-2202-9-117 PMC2637876

[16] Waghabi MC , de Souza EM , de Oliveira GM , *et al* Pharmacological inhibition of transforming growth factor beta signaling decreases infection and prevents heart damage in acute Chagas’ disease. Antimicrob Agents Chemother. 2009; 53: 4694–701. 19738024 10.1128/AAC.00580-09 PMC2772341

[17] Fagarasan S , Kawamoto S , Kanagawa O , *et al* Adaptive immune regulation in the gut: T cell‐dependent and T cell‐independent IgA synthesis. Annu Rev Immunol. 2010; 28: 243–73. 20192805 10.1146/annurev-immunol-030409-101314

[18] Godínez‐Victoria M , Drago‐Serrano ME , Reyna‐Garfias H , *et al* Effects on secretory IgA levels in small intestine of mice that underwent moderate exercise training followed by a bout of strenuous swimming exercise. Brain Behav Immun. 2012; 26: 1300–9. 22884415 10.1016/j.bbi.2012.07.018

[19] Kawamoto S , Tran TH , Maruya M , *et al* The inhibitory receptor PD‐1 regulates IgA selection and bacterial composition in the gut. Science. 2012; 336: 485–9. 22539724 10.1126/science.1217718

[20] Shikina T , Hiroi T , Iwatani K , *et al* IgA class switch occurs in the organized nasopharynx‐ and gut‐associated lymphoid tissue, but not in the diffuse lamina propria of airways and gut. J Immunol. 2004; 172: 6259–64. 15128814 10.4049/jimmunol.172.10.6259

[21] Seo GY , Youn J , Kim PH . IL‐21 ensures TGF‐beta 1‐induced IgA isotype expression in mouse Peyer's patches. J Leukoc Biol. 2009; 85: 744–50. 19168593 10.1189/jlb.0708450

[22] Giannelli V , Di Gregorio V , Iebba V , *et al* Microbiota and the gut‐liver axis: bacterial translocation, inflammation and infection in cirrhosis. World J Gastroenterol. 2014; 20: 16795–810. 25492994 10.3748/wjg.v20.i45.16795 PMC4258550

[23] Peterson DA , McNulty NP , Guruge JL , *et al* IgA response to symbiotic bacteria as a mediator of gut homeostasis. Cell Host Microbe. 2007; 2: 328–39. 18005754 10.1016/j.chom.2007.09.013

[24] Fagarasan S , Kinoshita K , Muramatsu M , *et al* *In situ* class switching and differentiation to IgA‐producing cells in the gut lamina propria. Nature. 2001; 413: 639–43. 11675788 10.1038/35098100

[25] O'Hara AM , Shanahan F . The gut flora as a forgotten organ. EMBO Rep. 2006; 7: 688–93. 16819463 10.1038/sj.embor.7400731 PMC1500832

[26] Kinross J , von Roon AC , Penney N , *et al* The gut microbiota as a target for improved surgical outcome and improved patient care. Curr Pharm Des. 2009; 15: 1537–45. 19442171 10.2174/138161209788168119

[27] Mittal R , Coopersmith CM . Redefining the gut as the motor of critical illness. Trends Mol Med. 2014; 20: 214–23. 24055446 10.1016/j.molmed.2013.08.004 PMC3959633

[28] Shimizu K , Ogura H , Hamasaki T , *et al* Altered gut flora are associated with septic complications and death in critically ill patients with systemic inflammatory response syndrome. Dig Dis Sci. 2011; 56: 1171–7. 20931284 10.1007/s10620-010-1418-8 PMC3059822

[29] Zaborin A , Smith D , Garfield K , *et al* Membership and behavior of ultra‐low‐diversity pathogen communities present in the gut of humans during prolonged critical illness. MBio. 2014; 5: e01361–14. 25249279 10.1128/mBio.01361-14 PMC4173762

[30] Hayakawa M , Asahara T , Henzan N , *et al* Dramatic changes of the gut flora immediately after severe and sudden insults. Dig Dis Sci. 2011; 56: 2361–5. 21384123 10.1007/s10620-011-1649-3

[31] Kamada N , Seo SU , Chen GY , *et al* Role of the gut microbiota in immunity and inflammatory disease. Nat Rev Immunol. 2013; 13: 321–35. 23618829 10.1038/nri3430

[32] Stefanutti G , Pierro A , Parkinson EJ , *et al* Moderate hypothermia as a rescue therapy against intestinal ischemia and reperfusion injury in the rat. Crit Care Med. 2008; 36: 1564–72. 18434898 10.1097/CCM.0b013e3181709e9f

[33] Kim M , Park SW , Kim M , *et al* Isoflurane post‐conditioning protects against intestinal ischemia‐reperfusion injury and multiorgan dysfunction *via* transforming growth factor‐β1 generation. Ann Surg. 2012; 255: 492–503. 22266638 10.1097/SLA.0b013e3182441767 PMC3288790

[34] Takahashi E , Kataoka K , Indalao IL , *et al* Oral clarithromycin enhances airway immunoglobulin A (IgA) immunity through induction of IgA class switching recombination and B‐cell‐activating factor of the tumor necrosis factor family molecule on mucosal dendritic cells in mice infected with influenza A virus. J Virol. 2012; 86: 10924–34. 22896605 10.1128/JVI.01207-12 PMC3457149

[35] Sawabuchi T , Suzuki S , Iwase K , *et al* Boost of mucosal secretory immunoglobulin A response by clarithromycin in paediatric influenza. Respirology. 2009; 14: 1173–9. 19909463 10.1111/j.1440-1843.2009.01639.x

[36] Cerutti A , Rescigno M . The biology of intestinal immunoglobulin A responses. Immunity. 2008; 28: 740–50. 18549797 10.1016/j.immuni.2008.05.001 PMC3057455

[37] Yoshiya K , Lapchak PH , Thai TH , *et al* Depletion of gut commensal bacteria attenuates intestinal ischemia/reperfusion injury. Am J Physiol Gastrointest Liver Physiol. 2011; 301: G1020–30. 21903760 10.1152/ajpgi.00239.2011

[38] Palm NW , de Zoete MR , Cullen TW , *et al* Immunoglobulin A coating identifies colitogenic bacteria in inflammatory bowel disease. Cell. 2014; 158: 1000–10. 25171403 10.1016/j.cell.2014.08.006 PMC4174347

[39] Mathias A , Longet S , Corthésy B . Agglutinating secretory IgA preserves intestinal epithelial cell integrity during apical infection by Shigella flexneri. Infect Immun. 2013; 81: 3027–34. 23753631 10.1128/IAI.00303-13 PMC3719585

[40] Suzuki K , Meek B , Doi Y , *et al* Aberrant expansion of segmented filamentous bacteria in IgA‐deficient gut. Proc Natl Acad Sci USA. 2004; 101: 1981–6. 14766966 10.1073/pnas.0307317101 PMC357038

[41] Peng X , Cebra JJ , Adler MW , *et al* Morphine inhibits mucosal antibody responses and TGF‐beta mRNA in gut‐associated lymphoid tissue following oral cholera toxin in mice. J Immunol. 2001; 167: 3677–81. 11564781 10.4049/jimmunol.167.7.3677

[42] Li L , Zhao XY , Wang BE . Down‐regulation of transforming growth factor beta 1/activin receptor‐like kinase 1 pathway gene expression by herbal compound 861 is related to deactivation of LX‐2 cells. World J Gastroenterol. 2008; 14: 2894–9. 18473417 10.3748/wjg.14.2894 PMC2710734

[43] Yamada Y , Mashima H , Sakai T , *et al* Functional roles of TGF‐β1 in intestinal epithelial cells through Smad‐dependent and non‐Smad pathways. Dig Dis Sci. 2013; 58: 1207–17. 23306843 10.1007/s10620-012-2515-7

[44] Inman GJ , Nicolás FJ , Callahan JF , *et al* SB‐431542 is a potent and specific inhibitor of transforming growth factor‐beta superfamily type I activin receptor‐like kinase (ALK) receptors ALK4, ALK5, and ALK7. Mol Pharmacol. 2002; 62: 65–74. 12065756 10.1124/mol.62.1.65

[45] Stavnezer J , Kang J . The surprising discovery that TGF beta specifically induces the IgA class switch. J Immunol. 2009; 182: 5–7. 19109126 10.4049/jimmunol.182.1.5

