# Supplementary material for: TGF‐β1 improves mucosal IgA dysfunction and dysbiosis following intestinal ischaemia–reperfusion in mice
Source: J Cell Mol Med. 2016 Jan 28;20(6):1014–23. doi: 10.1111/jcmm.12789 (PMC4882980; doi:10.1111/jcmm.12789)
Supplement: Supplementary file 1 — Data S1 Materials and methods. [file JCMM-20-1014-s001.docx]

**Materials and Methods**

*Animals and Operative Procedure*

The current experiment was approved by the Animal Care Committee of Sun Yat-sen University, GuangDong, China, and was performed in accordance with National Institutes of Health guidelines for the use of experimental animals. 8-10 wks old specific pathogen-free male Balb/c mice (28.6-32.3g) were housed in individual cages in a temperature-controlled room with alternating 12 h light:dark cycles, and acclimated for 1 wk before protocol entry.

Mice were anesthetized with pentobarbital by intraperitoneal injection (70mg/kg at induction and 30mg/kg at 1 h later) and ketamine (50mg/kg at induction). The small intestine was exteriorized by midline laparotomy, and the intestinal I/R injury was established by occluding the superior mesenteric artery (SMA) with a microvessel clip for 1 h, as we described previously. After 1 h of ischemia, the clip was removed and abdominal incision was sutured. During the study period, all procedures were performed on spontaneously breathing animals, and body temperature was maintained at about 36°C with the aid of a heating pad.

*Groups and Drug Administration*

The current experimental protocol was determined according to previous literatures and our preliminary results. The mice were randomly allocated into 5 groups, as follows. Sham group (Sham): the animals received adjuvants and underwent laparotomy with isolation of the SMA, but the SMA was not occluded; I/R group (Injury): the animals received adjuvants, and intestinal I/R was induced by clamping the SMA for 1 h followed by declamping; TGF-β1 group (TGF): recombinant human TGF-β1 (PeproTech Inc, Rocky Hill, NJ) was reconstituted in 10mM citric acid (pH 3.0) and then diluted in PBS containing 5% trehalose (pH 7.2), according to the manufacture’s instruction. Single TGF-β1 1μg (100μl) was infused intravenously through caudal vein at 15 min before the beginning of ischemia. TGF-β1 inhibitor group (SB): SB-431542, a potent and specific inhibitor of TGF-β receptor I, was dissolved in 10% dimethyl sulfoxide (DMSO) at 25°C. Single SB-431542 0.5mg (100μl) was injected intraperitoneally at 30 min before the beginning of ischemia; TGF-β1+SB-431542 group (TGF+SB): isometric TGF-β1 and SB-431542 was administrated at corresponding time point, respectively. The detailed experimental protocol was shown in Fig. 1.

*Biological samples collection*

After euthanizing the mice, a ~0.5cm segment of intestine was cut from 10cm to terminal ileum for immunohistochemical analysis. PP were carefully excised from the serosal side of the small intestine. After removal of PP, the intestine was opened longitudinally on ice and the fecal contents were collected for detecting IgA-binding bacteria. An intestinal fragment (10cm in length) next to ileocecal valve was flushed with a total of 3ml of chilled PBS solution, and the intestinal washings were collected for detecting sIgA concentration. Then this fragment was cut into small pieces (∼5 mm in length) for isolating cells from intestinal LP ^[26]^. Another segment of small intestine (10cm) was obtained from 12 cm to terminal ileum, and washed with cold saline. The intestinal mucosa was scraped off gently from this segment, dried with suction paper, and preserved at liquid nitrogen for detecting IgA mRNA expression ^[27]^. Finally, cecal feces were collected for analyzing bacterial communities in the gut. For each parameter, 5 to 6 samples per group were collected and analyzed.

*Immunohistochemical analysis*

The small intestine segment ~0.5cm was promptly fixed in 4% paraformaldehyde and embedded in paraffin. The fixed tissues were sectioned transversely in 4μm sections, and stained with hematoxylin-eosin, and then examined by light microscope. Two independent and blinded pathologists evaluated the damage of intestinal mucosa by using Chiu’s score, as described previously. A minimum of five randomly chosen fields from each sample were evaluated and averaged to determine mucosal injury.

To perform immunofluorescence staining, the paraffin sections of small intestines and PP were firstly subjected to deparaffinization and rehydration, and endogenous peroxidase activity was quenched with 3% H_2_O_2_. The antigen retrieval was performed by using sodium citrate buffer. Then the tissue was blocked with normal goat serum in PBS (5%) for 30 min in a humidifying chamber and rinsed three times in PBS for 5 min each. For detection of IgA expression, the sections were stained with FITC-conjugated anti-mouse IgA diluted 1/100 (559354, BD Pharmingen, San Diego, CA) for 1 h at room temperature in the dark. For detection of AID expression, the sections of PP and small intestine were stained with anti-AID antibody diluted 1/100 (ab93596, Abcam Inc, Cambridge, MA). At the end, nuclear staining was accomplished with 4’, 6-diamidino-2-phenylindole (DAPI) and mounted with Fluoromount-G (Southern Biotechnology Associates, Birmingham, AL). The stained slides were examined with a Nikon fluorescence microscope.

*Cells isolation from the PP and LP*

Cells suspension from the PP and LP were prepared according to the modified method descried previously. In brief, PP were gently washed by PBS and pressed through a steel mesh grid. Then the PP fragments were incubated with collagenase VIII (40 U/mL; Sigma, St. Louis, MO) in RPMI 1640 medium for 1 h at 37°C with constant stirring at 100 rpm. The cell suspensions were collected from the supernatant and passed through 100 micron nylon filters to remove debris and connective tissue.

For isolation cells from the LP, the small pieces of intestine were incubated incubated in PBS containing 2 mM EDTA-Na_2_, 1 mM dithiothreitol and 5% fetal bovine serum (FBS) at 37°C for 15 min on a shaker at 150 rpm. Supernatants containing epithelial and intraepithelial cells were removed. The remaining tissues were incubated with RPMI 1640 medium containing collagenase VIII (100 U/mL), 5% FBS, glutamine, and a 1% antibiotic mixture (penicillin and streptomycin) at 37°C for 45 min at 150 rpm in a water shaker. Supernatants containing LP cells were filtered through 100 micron nylon filters. Suspensions were then centrifuged at 1500 rpm for 10min at 4°C, the cell pellets were resuspended in 40% Percoll (Sigma, St. Louis, MO), and the cell suspensions were overlaid on 80% Percoll. After centrifugation at 1800 rpm for 20 min at 25°C, lymphocytes suspensions were aspirated from the interface of 40/80% Percoll and diluted with RPMI 1640. The suspensions were centrifuged at 1400 rpm for 5 min at 4°C and removed the supernatants. Viable cells were counted by using trypan blue dye exclusion.

*Quantitative real-time PCR (**qRT-PCR) and Flow Cytometry*

By using qRT-PCR and flow cytometric analysis, mRNA expression of biomarkers and percentage of IgA^+^/IgM^+^B220^+^ cells were applied to investigate IgA class switching.

Total RNA from the cells and the mucosal tissues was isolated using TRIzol reagent (Invitrogen, Carlsbad, CA) according to the manufacturer's protocol. RNA samples were treated with DNase I (Invitrogen, Carlsbad, CA) and then quantified by spectrophotometer and 1% agarose gel electrophoretic processing. RNA for reverse-transcription with random hexamers were used for first-strand cDNA synthesis. All procedures were carried out according to the manufacturer's instructions. And qRT-PCR was performed on a C1000 Touch Thermal cycler (Bio-Rad laboratories Inc, Hercules, CA) featuring a reaction mixture with SYBR Green as fluorescent dye. TGF-β1 and activation-induced cytidine deaminase (AID) are essential to IgA CSR, and germline α transcripts (GLTα) and Iμ-Cα circle transcripts (αCTs) are the representative activation biomarkers for IgA switching. The primers for TGF-β1, AID, GLTα, αCTs, IgA and glyceraldehyde-3-phosphate dehydrogenase (GAPDH) were determined based on previous reports (Table 1). The relative amounts of mRNA transcripts were calculated by using the standard curve method, and normalized by control GAPDH.

Flow cytometric analysis was performed according to the modified method descried previously. Briefly, the cell pellets obtained from PPs and LP were resuspended in cold Stain Buffer containing FBS (554656, BD Pharmingen, San Diego, CA). The lymphocytes from PPs and LP were stained with FITC-conjugated anti-mouse IgA (559354, BD Pharmingen), PE-conjugated anti-mouse IgM (553409, BD Pharmingen), PE-Cy™7 conjugated anti-mouse CD45R/B220 (552772, BD Pharmingen) to identify IgA^+^ and IgM^+^B220^+^ in the PP and LP, respectively. All antibodies were diluted to 1μg/ml in Stain Buffer. Incubations were carried out for 30 min on ice protected from light. Following staining, the cells were washed twice in Stain Buffer and then detected with a FACSCalibur flow cytometer (BD Biosciences, San Jose, CA). The data was analyzed by Flowjo software.

*Detection of sIgA in intestinal lavage fluid*

SIgA concentration was detected in the intestinal washings by using the ELISA assay (Cusabio, Wuhan, China), as described previously. Briefly, the washout fluid was centrifuged at 4000 rpm for 20 min at 4°C, and then the supernatant was harvested to measure the sIgA concentration according to the manufacturer's instruction.

*Gut bacterial community analysis*

Gut microbiota was analyzed by a high-throughput 16S rRNA gene sequencing on the Illumina MiSeq platform. The compositions of microbial communities were assessed according to the amplification and sequencing protocol of Miseq system. Briefly, bacteria genomic DNA was extracted from 0.5g cecal feces through PowerSoil® DNA Isolation Kit (MO BIO Laboratories, Carlsbad, CA). The primers of V4 hypervariable regions were designed with overhang adapters, and used to amplify templates from genomic DNA. A subsequent limited-cycle amplification step was performed to attach indicies and Illumina sequencing adapters by the Nextera® XT Index kit. Libraries were normalized, pooled, and then sequenced on the sequencing protocol of the Miseq system. Finally, data analysis was performed by Mothur software.

*Evaluation of intestinal* *bacteria coated with IgA*

Flow cytometric analysis of IgA-binding bacteria was performed as previously described. In brief, feces collected from intestine were suspended in PBS (100 μl to 10 mg feces), homogenized well and centrifuged at 1400 rpm for 5 min to remove larger particle from bacteria. Supernatant was centrifuged at 8500 rpm for 10 min to remove non-bound Igs. Then the pellets were resuspended in 1 ml of FBS/PBS (1% w/v). Bacteria were stained with FITC-conjugated anti-mouse IgA (559354, BD Pharmingen, San Diego, CA) on ice for 20 min and washed with PBS. Finally, bacteria pellets were resuspended in 4 μg/ml propidium iodide (PI, Sigma, St. Louis, MO)/PBS, and analyzed by FACScalibur. All events that stained with PI were regarded as bacteria. The percentage of fecal bacteria coated with IgA was calculated as: the number of PI^+^ IgA^+^ cells/the number of total PI^+^ cells×100.

*Survival Analysis*

The survival analysis was performed in other independent mice as we previously described. Briefly, the mice receiving the same interventions were used to detect survival time and mortality rate. After ischemia, the animals were transferred to their individual cages and allowed free access to water. Each mouse was monitored via video recording for 24 h.

*Statistical Analysis*

The data were analyzed with SPSS 15.0 software (SPSS Inc, Chicago, IL). Survival time after reperfusion was expressed as median (95% confidence intervals), and results were compared by Kaplan–Meier log-rank test. The other data were expressed as mean±SE, and one-way ANOVA (Tukey posttest) was used for comparisons. *P*<0.05 in two-tailed testing was considered statistically significant.
